# Supplementary material for: The NAD salvage pathway in mesenchymal cells is indispensable for skeletal development in mice
Source: Nat Commun. 2023 Jun 17;14:3616. doi: 10.1038/s41467-023-39392-7 (PMC10276814; doi:10.1038/s41467-023-39392-7)
Supplement: Supplementary file 1 — Supplementary Information [file 41467_2023_39392_MOESM1_ESM.pdf]

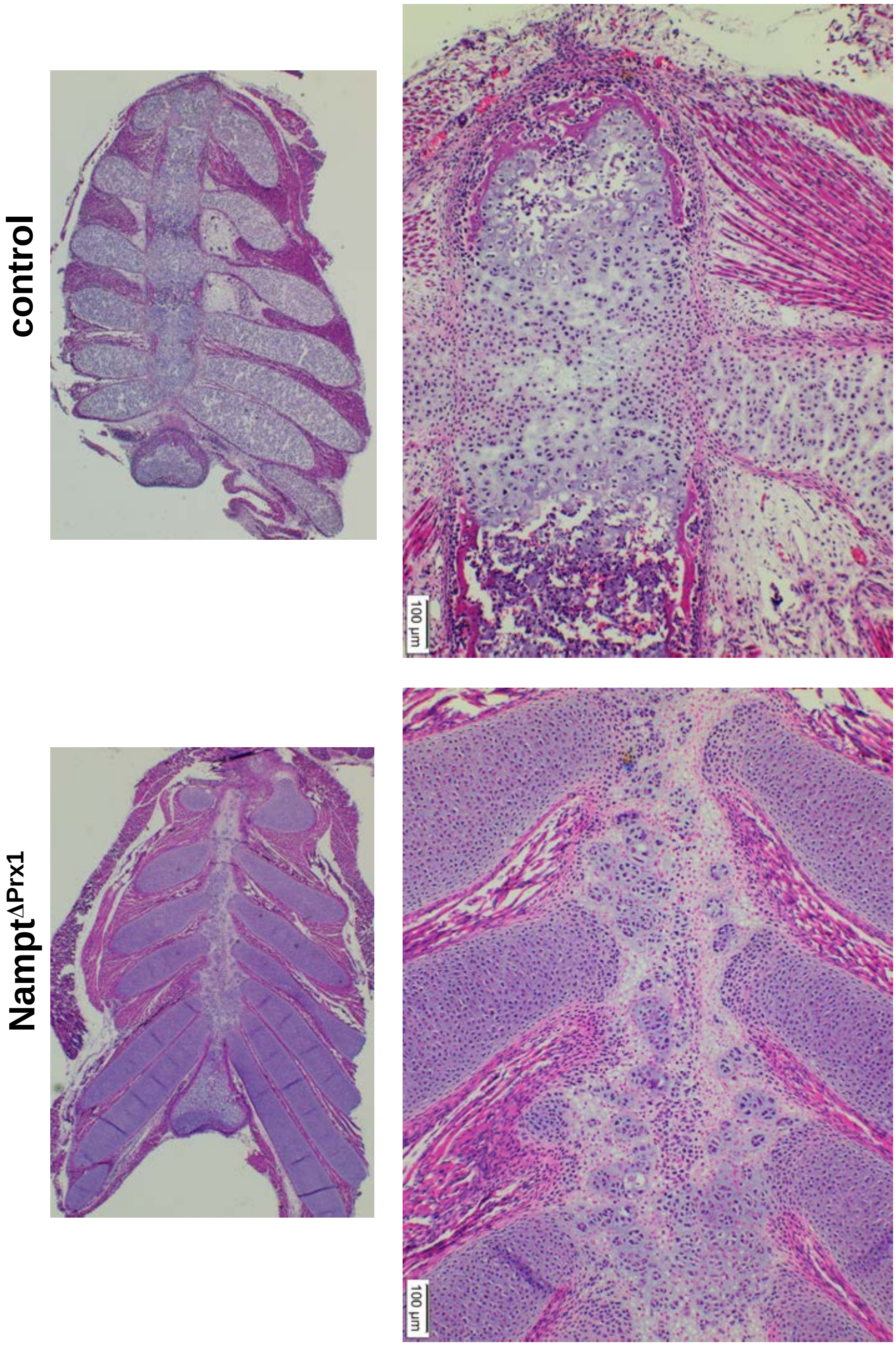

Supplementary Figure 1. Nampt is essential for proper formation of the sternum. H&E staining of histological sections of P2 Nampt$^{f/+}$ (control) and Nampt$^{f/f}$;Prx1-Cre (Nampt$^{\Delta Prx1}$) sternum (n=3).

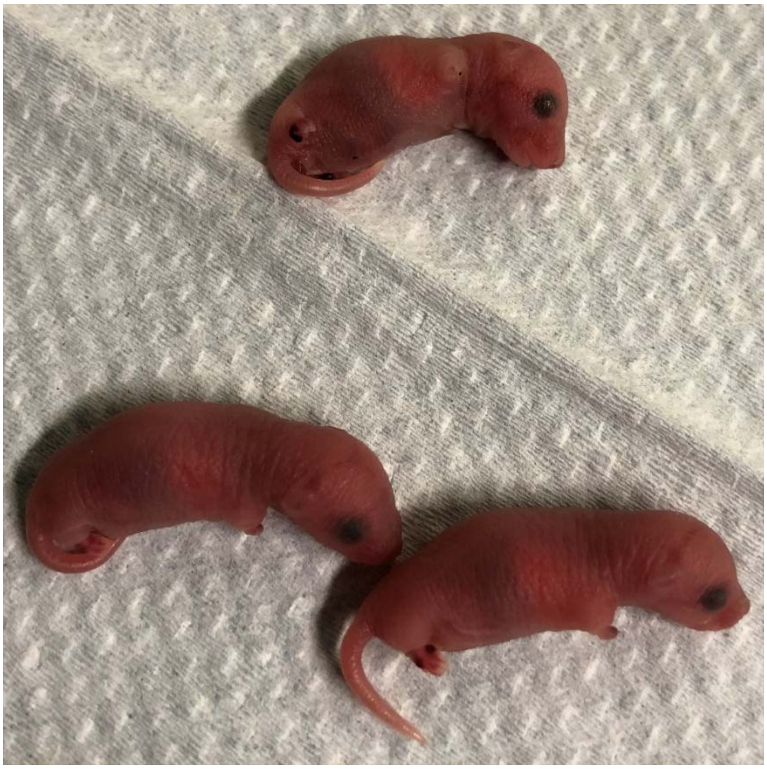

Supplementary Figure 2. A low dose of NR did not attenuate the limb defects in mice lacking Nampt. Nampt$^{f/f}$;Prx1-Cre (Nampt$^{\Delta Prx1}$) mice born from dams supplemented with 400 mg/kg NR during gestation. Two independent litters of mice were obtained showing similar effects.

Control + pNR

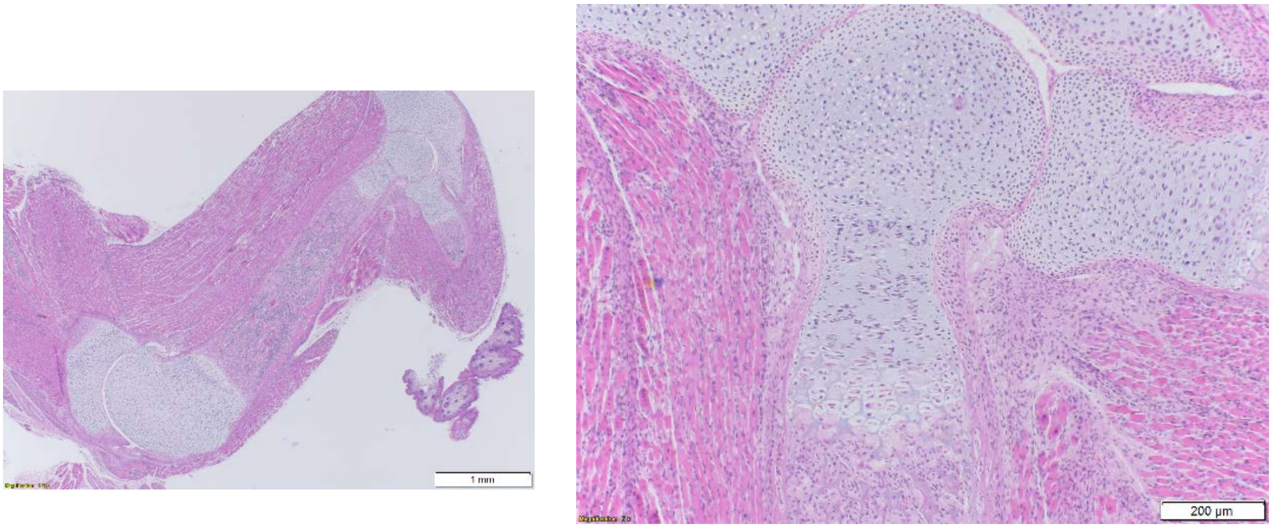

Nampt$^{\Delta Prx1}$ + pNR

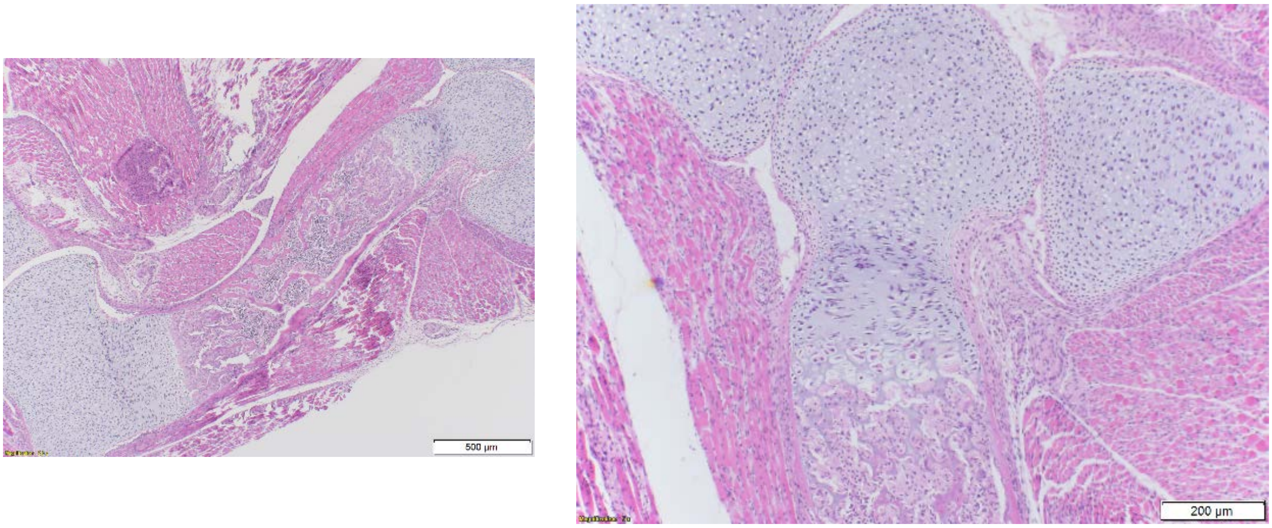

Supplementary Figure 3. NR administration during pregnancy rescued most of the skeletal defects caused by Nampt deletion. NR was administered in the drinking water to dams during pregnancy (pNR). H&E staining of histological sections of the whole (left) and distal humerus (right) of Nampt$^{f/+}$ (control) and Nampt$^{f/f}$;Prx1-Cre (Nampt$^{\Delta Prx1}$) at P2. Three independent litters of mice were obtained and showed similar effects.

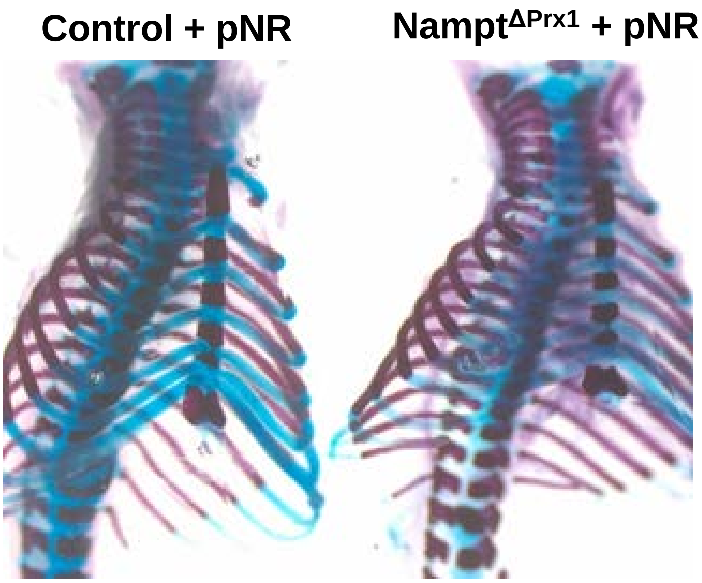

Supplementary Figure 4. NR administration during pregnancy rescued most of the skeletal defects caused by Nampt deletion. (A) NR was administered in the drinking water to dams during pregnancy (pNR). Whole-mount alcian blue-alizarin red S staining of ribcage and sternum of P2 $Nampt^{f/+}$ (control) and $Nampt^{f/f}$;Prx1-Cre ($Nampt^{\Delta Prx1}$).

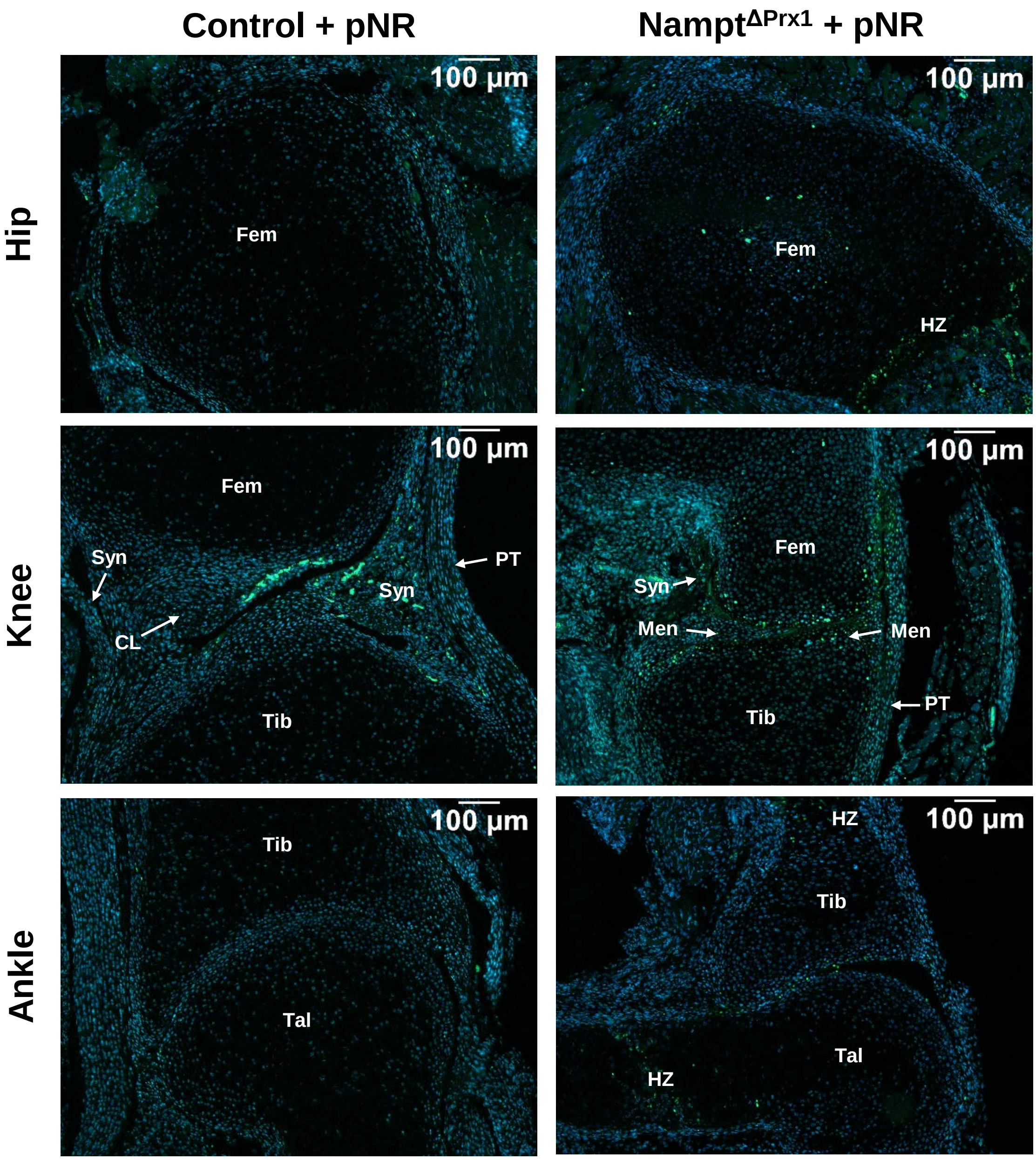

Supplementary Figure 5. TUNEL staining reveals increased cell death within the Nampt$^{\Delta Prx1}$ joints soon after cessation of NR. Sections from P2 Nampt$^{f/+}$ (control) and Nampt$^{f/f}$;Prx1-Cre (Nampt$^{\Delta Prx1}$) knees (representative of 2-3 pups per genotype) showing TUNEL staining (green) with DAPI counterstain (blue). Fem = femur; Tib = tibia; Men = meniscus; Syn = synovium; CL = cruciate ligament; PT = patellar tendon; Tal = talus; HZ = hypertrophic zone.

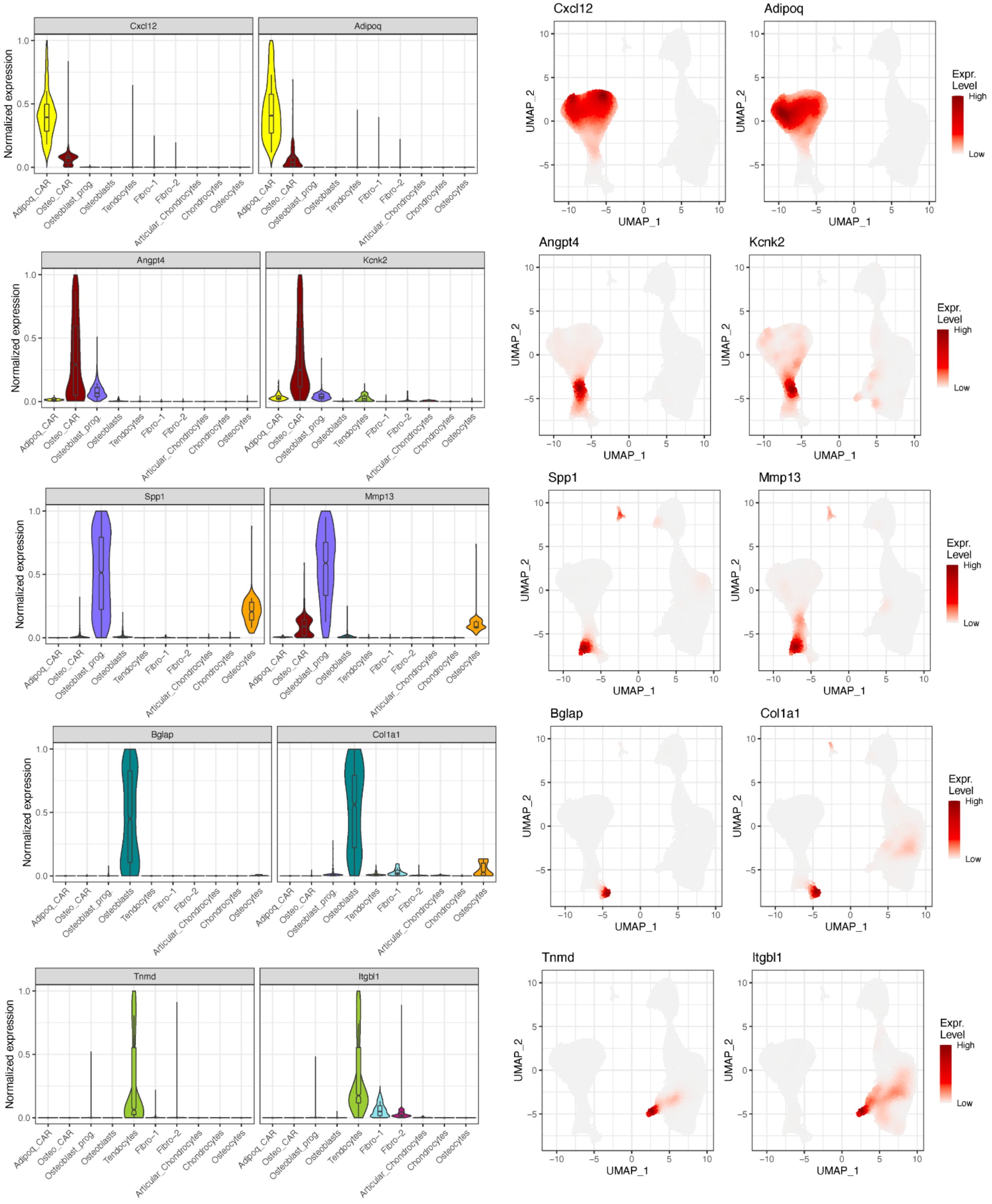

Supplementary Figure 6

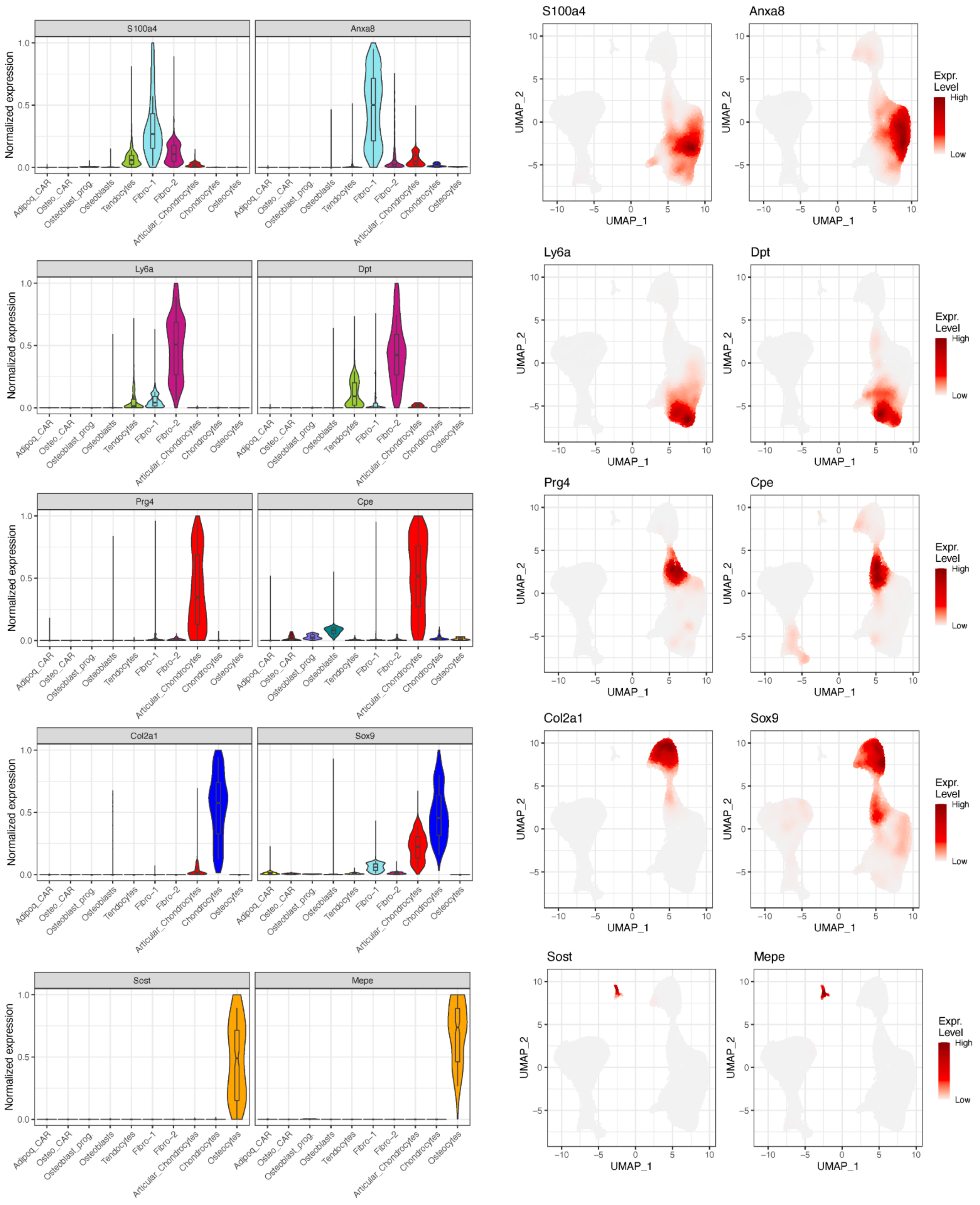

Supplementary Figure 6 (continued)

Supplementary Figure 6. Gene markers for mesenchymal bone cell clusters of published datasets. Expression levels of marker genes within each of major classes of non-hematopoietic cells (femoral bone and bone marrow fractions) based on two published single cell RNA sequencing datasets. UMAP-based clusters are depicted in Figure 6A. The box plots show maximum, first quartile, median, third quartile, and minimum values. Adipoq_CAR (n=15557 cells), Osteo_CAR (n=2210 cells), Osteoblast_prog. (n=1216 cells), Osteoblasts (n=683 cells), Tenocytes (n=1609 cells), Fibro-1 (n=8425 cells), Fibro-2 (n=4263 cells), Articular_Chondrocytes (n=3452 cells), Chondrocytes (n=4233 cells), Osteocytes (n=200 cells).

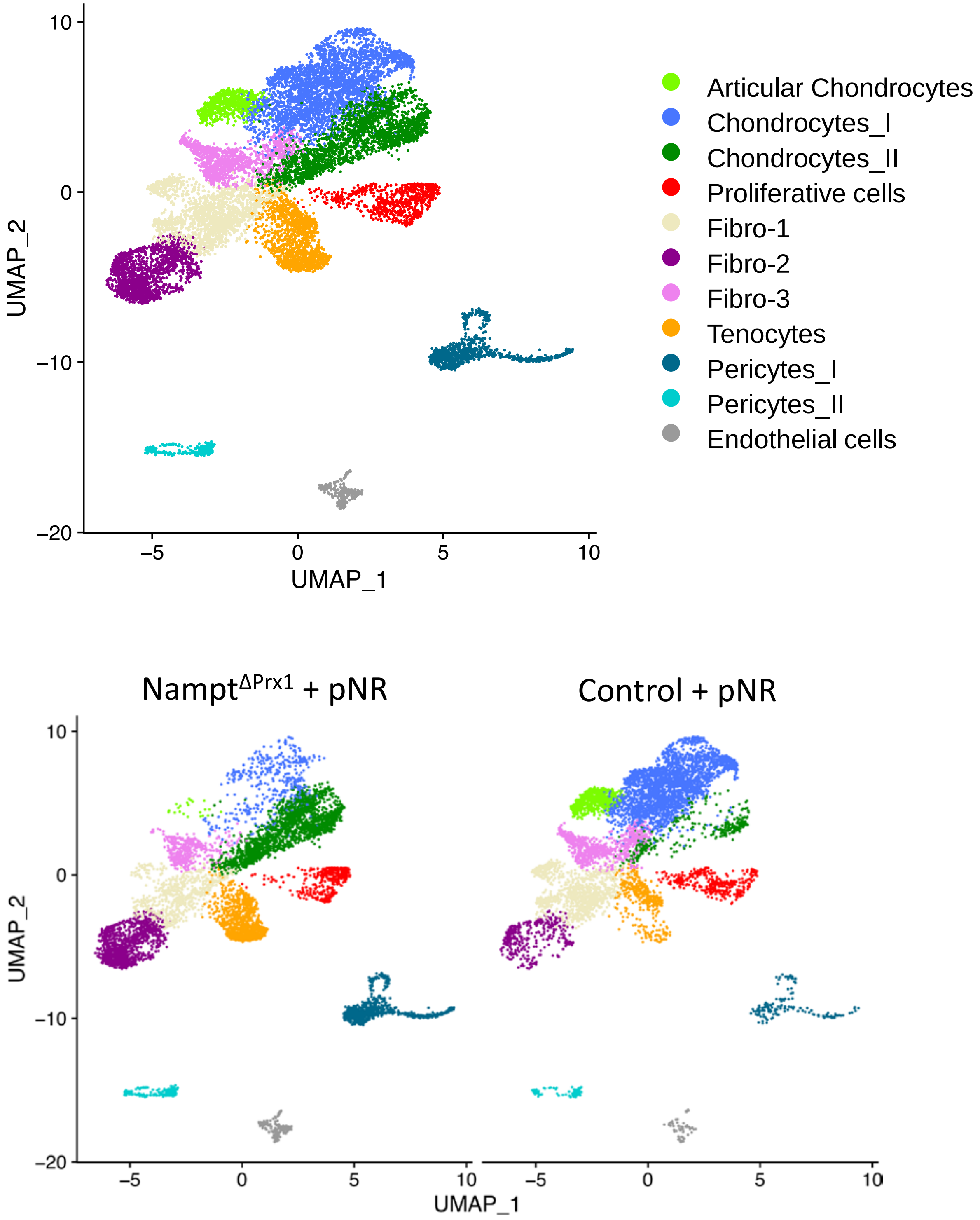

Supplementary Figure 7. Cell clusters obtained from femur and tibia growth plates. NR was administered in the drinking water to dams during pregnancy (pNR). Single cell RNA-seq analysis of cells extracted from the growth plates of P2 Nampt$^{\Delta Prx1}$ and littermate control mice. UMAP plot of chondrocytes and other mesenchymal linage cells obtained from mice of both genotypes (top) and each genotype separately (bottom).

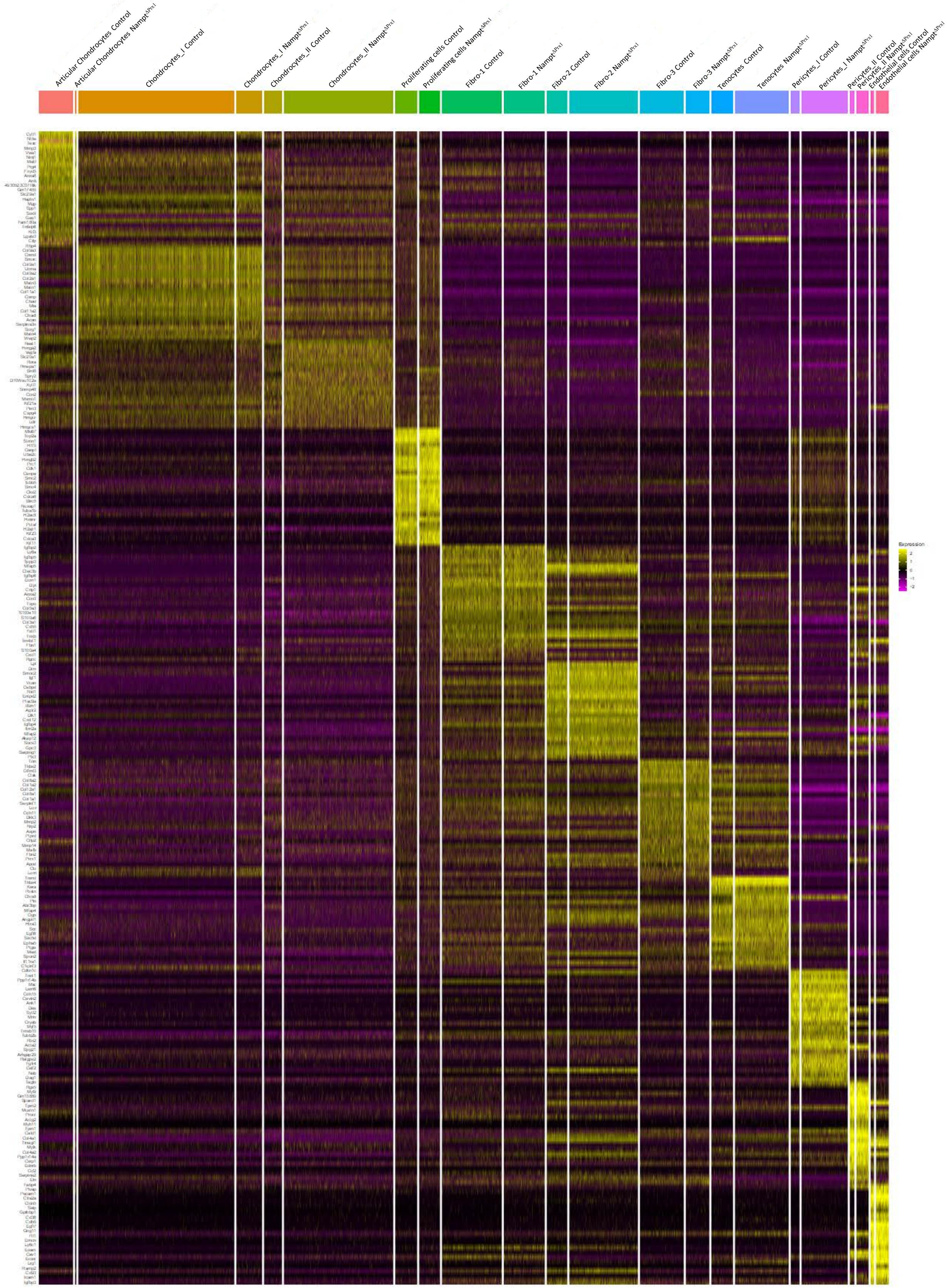

Supplementary Figure 8. Marker genes for clusters obtained from femur and tibia growth plates. Heatmap of top 25 marker genes that were differentially expressed in each cluster of cells extracted from the growth plates of P2 Nampt$^{\Delta Prx1}$ and control mice. UMAP-based clusters are depicted in Supplementary Figure 6 and Figure 8D.

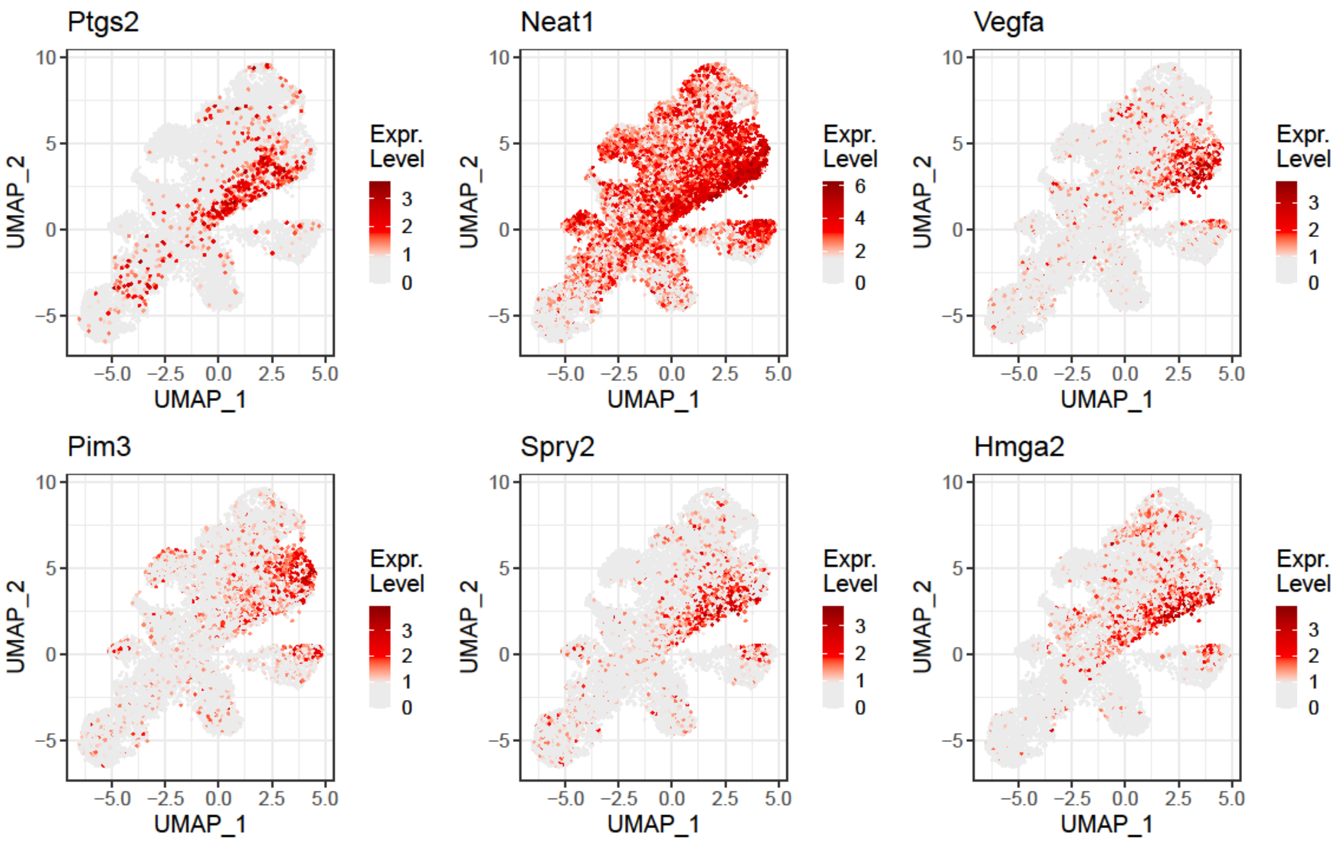

Supplementary Figure 9. Expression of genes upregulated in chondrocytes II versus chondrocytes I. Expression level of some of the genes increased in chondrocytes from Nampt$^{\Delta Prx1}$ mice (chondrocytes II). UMAP-based clusters are depicted in Fig. 8A.

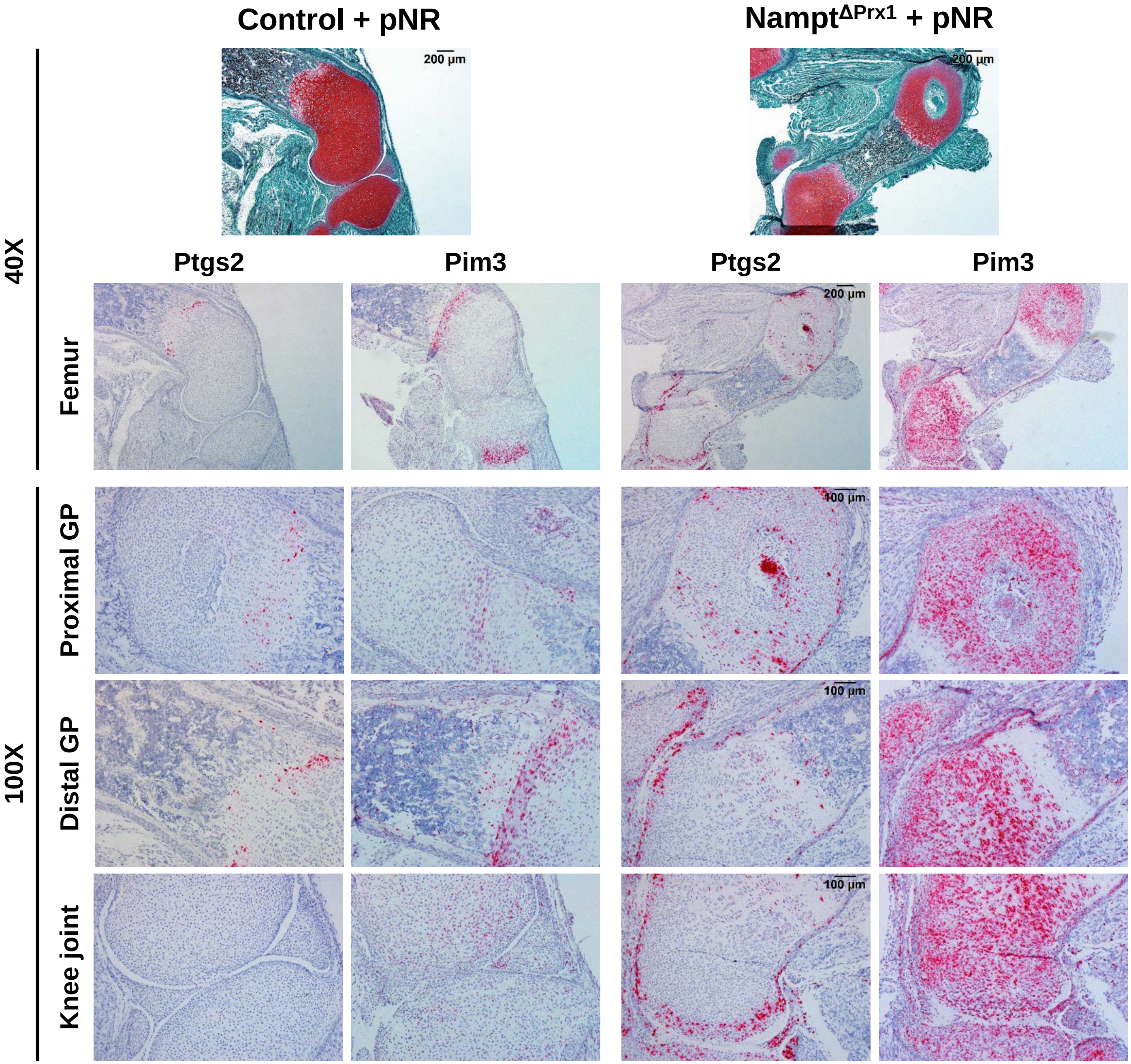

Supplementary Figure 10. Nampt deletion increases expression of Ptgs2 and Pim3 within the growth plate. NR was administered in the drinking water to dams during pregnancy (pNR). Sections from P2 $Nampt^{f/+}$ (control) and $Nampt^{f/f}$;Prx1-Cre ($Nampt^{\Delta Prx1}$) knees (representative of 2-3 pups per genotype) showing *in situ* hybridization for *Ptgs2* and *Pim3* within the femoral growth plates (GP) and knee joints.

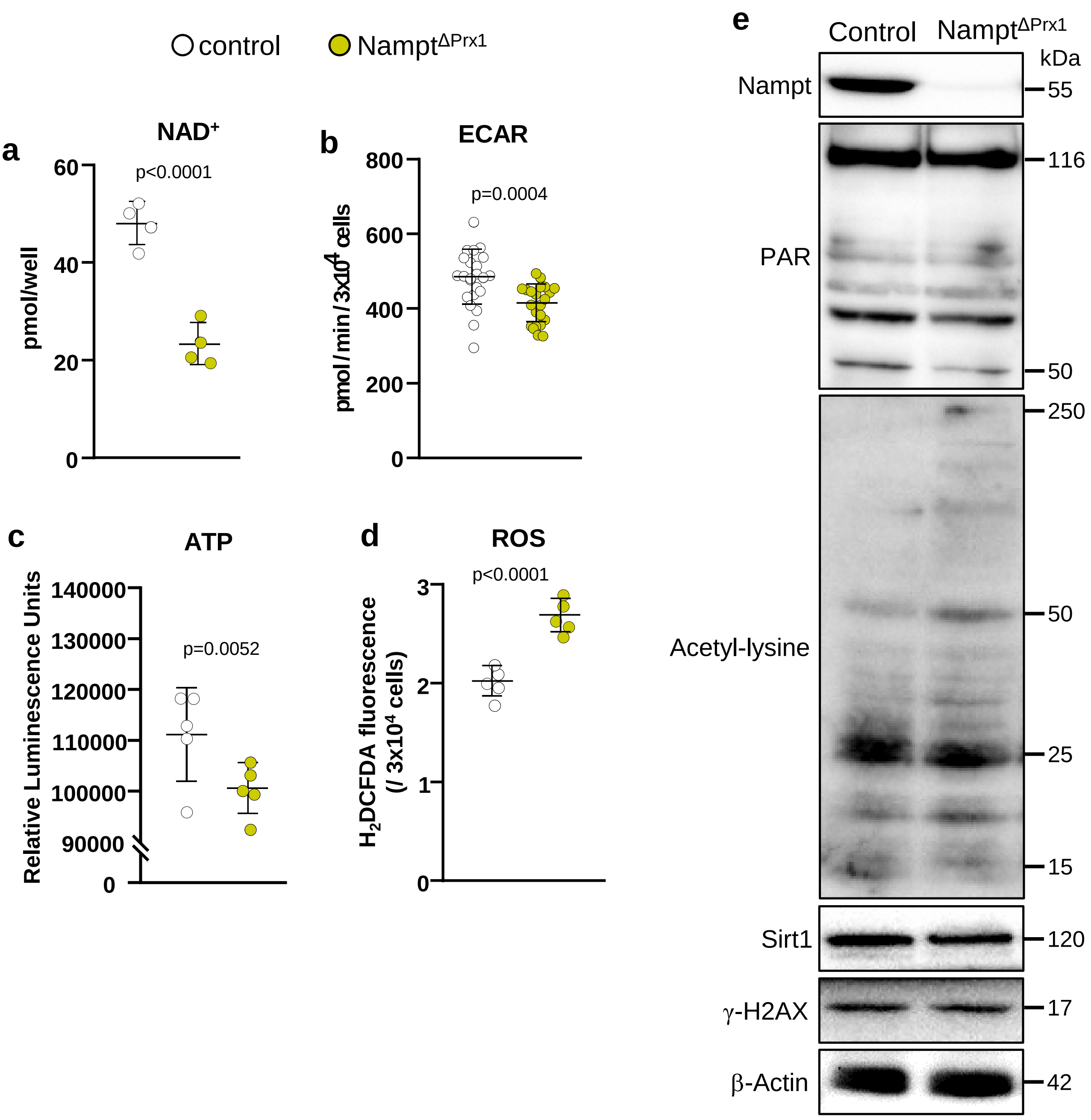

Supplementary Figure 11. Chondrocyte cultures from Nampt$^{\Delta Prx1}$ mice exhibit metabolic defects. Primary chondrocytes from two of each control and Nampt$^{\Delta Prx1}$ mice were pooled and cultured in hypoxia (1% O_2) for a total of 4 days. Measurements of intracellular NAD^+ (n=4 wells) (a); extracellular acidification rate (ECAR), an indicator of glycolysis (n=23 wells) (b); ATP (n=5 wells) (c); and reactive oxygen species (ROS) (n=5 wells) (d). Cell lysates were collected and (e) Western Blot performed for the indicated proteins. Graphs depict representative experiments. Each dot (a-d) represents one replicate well. Each experiment was repeated twice. Bars represent mean and SD. P values calculated by two-sided t-test.

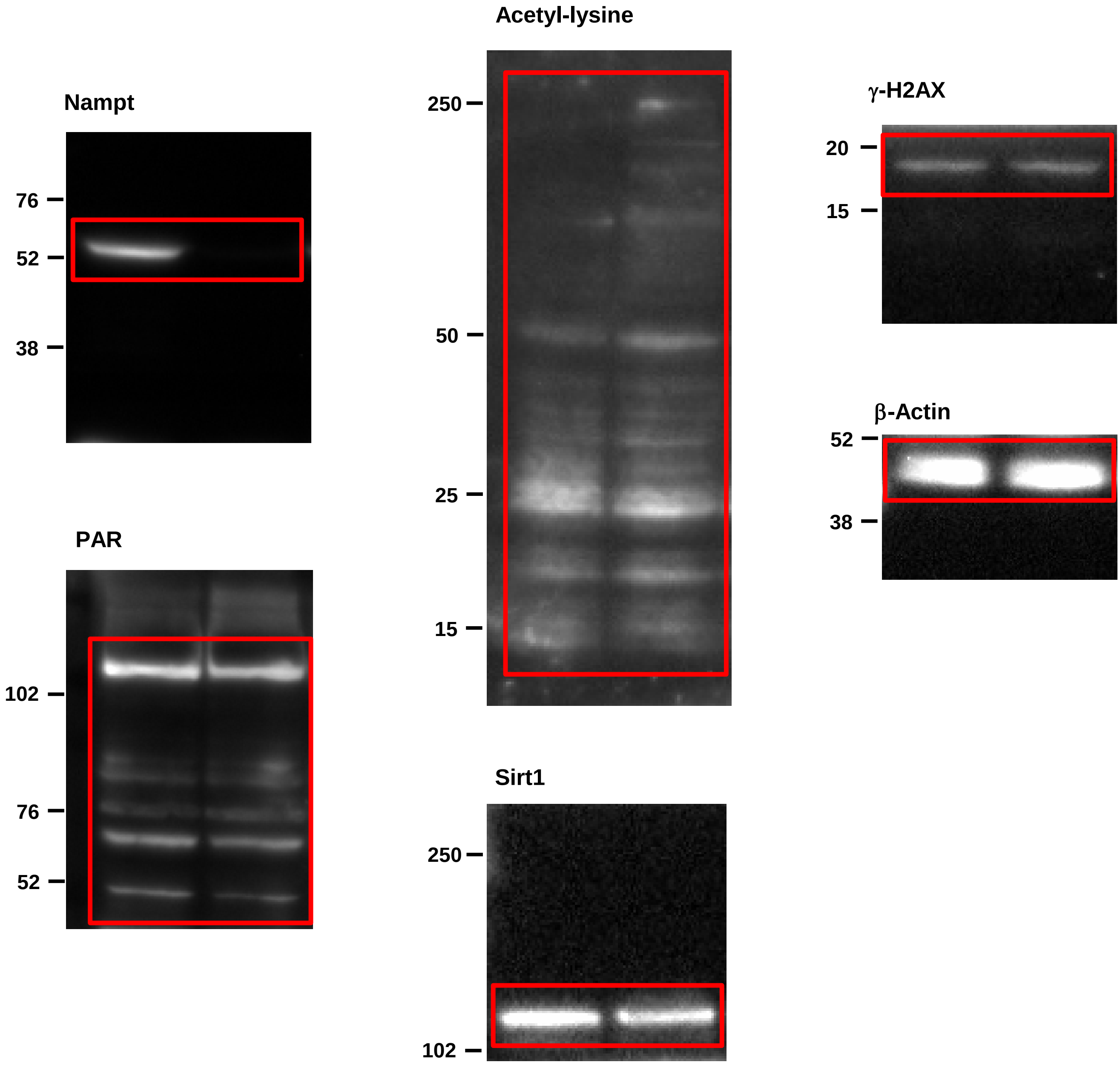

Supplementary Figure 12. Unprocessed scans of the Western blots shown in supplementary figure 11E.
