# Supplementary material for: The NAD salvage pathway in mesenchymal cells is indispensable for skeletal development in mice
Source: Nat Commun. 2023 Jun 17;14:3616. doi: 10.1038/s41467-023-39392-7 (PMC10276814; doi:10.1038/s41467-023-39392-7)
Supplement: Supplementary file 3 — Description of Additional Supplementary Files Document [file 41467_2023_39392_MOESM3_ESM.pdf]

Description of Additional Supplementary Files

Supplementary Data 1. Differential gene expression analysis between chondrocytes I and chondrocytes II.
