# Supplementary material for: The NAD salvage pathway in mesenchymal cells is indispensable for skeletal development in mice
Source: Nat Commun. 2023 Jun 17;14:3616. doi: 10.1038/s41467-023-39392-7 (PMC10276814; doi:10.1038/s41467-023-39392-7)
Supplement: Supplementary file 5 — Reporting Summary [file 41467_2023_39392_MOESM5_ESM.pdf]

Corresponding author(s): Maria Almeida

Last updated by author(s): Apr 27, 2023

Reporting Summary

Nature Portfolio wishes to improve the reproducibility of the work that we publish. This form provides structure for consistency and transparency in reporting. For further information on Nature Portfolio policies, see our Editorial Policies and the Editorial Policy Checklist.

Statistics

For all statistical analyses, confirm that the following items are present in the figure legend, table legend, main text, or Methods section.

n/a	Confirmed	
☐	☒	The exact sample size (*n*) for each experimental group/condition, given as a discrete number and unit of measurement
☐	☒	A statement on whether measurements were taken from distinct samples or whether the same sample was measured repeatedly
☐	☒	The statistical test(s) used AND whether they are one- or two-sided<br>*Only common tests should be described solely by name; describe more complex techniques in the Methods section.*
☐	☒	A description of all covariates tested
☐	☒	A description of any assumptions or corrections, such as tests of normality and adjustment for multiple comparisons
☐	☒	A full description of the statistical parameters including central tendency (e.g. means) or other basic estimates (e.g. regression coefficient) AND variation (e.g. standard deviation) or associated estimates of uncertainty (e.g. confidence intervals)
☐	☒	For null hypothesis testing, the test statistic (e.g. *F*, *t*, *r*) with confidence intervals, effect sizes, degrees of freedom and *P* value noted<br>*Give P values as exact values whenever suitable.*
☒	☐	For Bayesian analysis, information on the choice of priors and Markov chain Monte Carlo settings
☒	☐	For hierarchical and complex designs, identification of the appropriate level for tests and full reporting of outcomes
☒	☐	Estimates of effect sizes (e.g. Cohen's *d*, Pearson's *r*), indicating how they were calculated

Our web collection on statistics for biologists contains articles on many of the points above.

Software and code

Policy information about availability of computer code

Data collection	OsteoMeasure Analysis System (OsteoMetrics, Inc. Atlanta, GA); PIXImus densitometer (GE Lunar); micro-CT40 (Scanco Medical, Brüttiselen, Switzerland); Cytation 5 reader (BioTek Instruments, Winooski, VT, USA); Chromium Controller (10X Genomics, Pleasanton, CA) and Illumina NovaSeq 6000; QuantStudio3 (Applied Biosystems)
Data analysis	GraphPad Prism 8; CellRanger version 6.1.2 ; Seurat software version 4.1; Canonical Correlation Analysis; FindMarkersAll using MAST algorithm; Nebulosa package 1.8.0; mouse genome-scale metabolic model iMM1865 using piano R package 2.14.0

For manuscripts utilizing custom algorithms or software that are central to the research but not yet described in published literature, software must be made available to editors and reviewers. We strongly encourage code deposition in a community repository (e.g. GitHub). See the Nature Portfolio guidelines for submitting code & software for further information.

Data

Policy information about availability of data

All manuscripts must include a data availability statement. This statement should provide the following information, where applicable:

- Accession codes, unique identifiers, or web links for publicly available datasets
- A description of any restrictions on data availability
- For clinical datasets or third party data, please ensure that the statement adheres to our policy

The raw single cell RNA sequencing data are accessible in the NCBI's Sequence Read Archive (SRA) under BioProject PRJNA914642 [https://www.ncbi.nlm.nih.gov/bioproject/?term=PRJNA914642]. Quantitative data used in figure plots are provided within the Source Data file provided with this paper.

Research involving human participants, their data, or biological material

Policy information about studies with human participants or human data. See also policy information about sex, gender (identity/presentation), and sexual orientation and race, ethnicity and racism.

Reporting on sex and gender	Not applicable
Reporting on race, ethnicity, or other socially relevant groupings	Not applicable
Population characteristics	Not applicable
Recruitment	Not applicable
Ethics oversight	Not applicable

Note that full information on the approval of the study protocol must also be provided in the manuscript.

Field-specific reporting

Please select the one below that is the best fit for your research. If you are not sure, read the appropriate sections before making your selection.

☒ Life sciences ☐ Behavioural & social sciences ☐ Ecological, evolutionary & environmental sciences

For a reference copy of the document with all sections, see nature.com/documents/nr-reporting-summary-flat.pdf

Life sciences study design

All studies must disclose on these points even when the disclosure is negative.

Sample size	Sample size calculation was guided by previous studies in C57BL/6 mice using similar skeletal analysis, such that no sample size calculation was performed.
Data exclusions	No data was excluded from analysis.
Replication	In vitro experiments were repeated at least once, as specified in the figure legends.
Randomization	Assignment of animals to groups was decided by genotype and no treatments were performed on individual animals post-birth, thus randomization was not required.
Blinding	For the study of NamptΔPrx1 and littermate controls, limb size between genotypes was very different and did not allow for the analysis to be performed in a blind manner. For the study of NamptΔOsx1 and littermate controls, BMD and histomorphometric measurements were performed in a blinded manner.

Reporting for specific materials, systems and methods

We require information from authors about some types of materials, experimental systems and methods used in many studies. Here, indicate whether each material, system or method listed is relevant to your study. If you are not sure if a list item applies to your research, read the appropriate section before selecting a response.

Materials & experimental systems

n/a	Involved in the study
☐	☒ Antibodies
☒	☐ Eukaryotic cell lines
☒	☐ Palaeontology and archaeology
☐	☒ Animals and other organisms
☒	☐ Clinical data
☒	☐ Dual use research of concern
☒	☐ Plants

Methods

n/a	Involved in the study
☒	☐ ChIP-seq
☒	☐ Flow cytometry
☒	☐ MRI-based neuroimaging

Antibodies

Antibodies used

Antibodies used in western blot as follows: rabbit monoclonal antibody against Nampt (clone EPR21980, Abcam, ab236874, 1:1000), rabbit polyclonal antibody against acetylated-lysine (Cell Signaling, #9441, 1:1000); mouse monoclonal antibodies against PAR (clone 10H, Enzo Life Sciences, ALX-804-220-R100, 1:1000), Sirt1 (clone 1F3, Cell Signaling, #8469, 1:1000), phospho-Histone H2A.X (clone JBW301, Sigma-Aldrich, #05-636, 1:1000), and β-actin (clone ACTBD11B7, Santa Cruz Biotechnology, sc-81178, 1:2000). Secondary antibodies include: Mouse IgG1 binding protein (m-IgG1 BP) conjugated to Horseradish Peroxidase (HRP) (Santa Cruz, sc-525408, 1:2500); anti-rabbit IgG, HRP-linked Antibody (Cell Signaling, #7074, 1:1000)

Antibodies used in immunohistochemistry as follows: rabbit monoclonal antibody against Nampt (clone EPR21980, Abcam, ab236874, 1:3,000) or isotype control (clone EPR25A, Abcam ab172730, 1:9,000), mouse monoclonal IgG1 against type II collagen (clone II-II6B3, Developmental Studies Hybridoma Bank, 1:1,000)

Validation

All antibodies were commercially available and characterized by the manufacturers online, as found at the following links: Nampt antibody (https://www.abcam.com/products/primary-antibodies/visfatin-antibody-epr21980-ab236874.html); Acetylated-Lysine antibody (https://www.cellsignal.com/products/primary-antibodies/acetylated-lysine-antibody/9441); PAR antibody (https://www.enzolifesciences.com/ALX-804-220/poly-adp-ribose-monoclonal-antibody-10h/); Sirt1 antibody (https://www.cellsignal.com/products/primary-antibodies/sirt1-1f3-mouse-mab/8469); phospho-Histone H2A.X antibody (https://www.sigmaaldrich.com/US/en/product/mm/05636); β-actin antibody (https://www.scbt.com/p/beta-actin-antibody-actbd11b7); Rabbit monoclonal IgG [EPR25A] (https://www.abcam.com/products/primary-antibodies/rabbit-igg-monoclonal-epr25a-isotype-control-ab172730.html); Type II collagen antibody (https://dshb.biology.uiowa.edu/II-II6B3); Mouse IgG1 binding protein (m-IgG1 BP) conjugated to Horseradish Peroxidase (HRP) (https://www.scbt.com/p/m-igg1-bp-hrp); Anti-rabbit IgG, HRP-linked Antibody (https://www.cellsignal.com/products/secondary-antibodies/anti-rabbit-igg-hrp-linked-antibody/7074)

Animals and other research organisms

Policy information about studies involving animals; ARRIVE guidelines recommended for reporting animal research, and Sex and Gender in Research

Laboratory animals

Multiple genetically-modified strains of mice, all on a C57BL/6 genetic background, were studied between P0 and P28.

Wild animals

No wild animals were used

Reporting on sex

In most studies, information on sex was not collected because the effects on the skeleton were sex independent.
In the studies of Figure 5F-G a sex based analysis was performed (male n=5-12, female n=8-10).

Field-collected samples

No samples were collected from the field

Ethics oversight

All animal work was preapproved and done in accordance with the UAMS Institutional Animal Care and Use Committee. All animal studies complied with the ethical regulations and humane endpoints according to the NIH Guide for the Care and Use of Laboratory Animals.

Note that full information on the approval of the study protocol must also be provided in the manuscript.
